# Synchronization by uncorrelated noise: interacting rhythms in interconnected oscillator networks

**DOI:** 10.1038/s41598-018-24670-y

**Published:** 2018-05-03

**Authors:** John Hongyu Meng, Hermann Riecke

**Affiliations:** Engineering Sciences and Applied Mathematics, Northwestern University, Evanston, IL 60208 USA

## Abstract

Oscillators coupled in a network can synchronize with each other to yield a coherent population rhythm. How do multiple such rhythms interact with each other? Do these collective oscillations synchronize like individual oscillators? We show that this is not the case: for strong, inhibitory coupling rhythms can become synchronized by noise. In contrast to stochastic synchronization, this new mechanism synchronizes the rhythms even if the noisy inputs to different oscillators are completely uncorrelated. Key for the synchrony *across* networks is the reduced synchrony *within* the networks: it substantially increases the frequency range across which the networks can be entrained by other networks or by periodic pacemaker-like inputs. We demonstrate this type of robust synchronization for different classes of oscillators and network connectivities. The synchronization of different population rhythms is expected to be relevant for brain rhythms.

## Introduction

Substantial progress has been made in the understanding of the collective dynamics of oscillators that are coupled in a network; particularly the conditions for their synchronization are quite well understood^1,2^. Synchronization is important in many technologically relevant systems^3–6^. It plays also a a central functional role in many biological systems like the heart^7^ and the suprachiasmatic nucleus of the brain, which controls the circadian rhythm^8^. In the brain coherent activity of large ensembles of neurons manifests itself in macroscopically observable rhythms, which have been found in many brain regions^9^. Among them the widely observed *γ*-rhythm (30–100 Hz), which may play an important role in the communication between brain areas^10–16^, has been studied particularly extensively^17–22^.

In synchronous regimes the collective oscillations constitute a rhythmic population activity of a whole network of oscillators and can be thought of as the dynamics of a single oscillator. Using a mean-field approach, this allows a first step towards the description of the interaction between multiple, interconnected such networks, which has, for instance, been used in ecological studies to capture the spatial interaction between different population oscillations^23^. Since such interconnected or modular networks are quite common^24,25^, the interaction between multiple networks, each supporting its own rhythm or collective oscillation, is of great interest.

For interconnected networks the stability of a globally synchronous state and its dependence on the connectivity within and between the networks has been studied using the master stability function^26,27^. In the limit of weak coupling, which allows a phase description of the oscillators in terms of variants of the Kuramoto model, interconnected networks have been investigated for weak heterogeneity and weak noise^28–31^. A feature shared by both these approaches is that the dynamics of the oscillators within each of the interconnected networks are quite homogeneous; in particular, all their oscillation amplitudes are very similar.

However, there are important, strong population rhythms in which the individual oscillation amplitudes fluctuate substantially and not all oscillators participate in each cycle of the collective oscillations. Neuronal *γ*-rhythms are a characteristic example^17^. This raises the question whether the interaction between interconnected networks can couple sufficiently strongly to the internal degrees of freedom of the individual networks to modify the internal workings of the collective oscillations. Can this induce qualitative changes in the ability of the collective oscillations of the different networks to synchronize with each other or to an external pacemaker? In investigating these questions we are particularly motivated by the ubiquitous *γ*-rhythms^22^ and their behavior-dependent coherence across different brain areas^13^ as well as the simultaneous observation of multiple, different *γ*-rhythms in a single brain area that is presumably modularly organized^32–36^.

**Figure 1:**
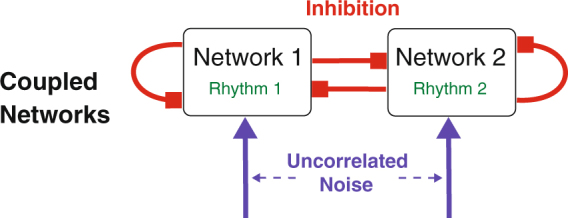
Interacting rhythms in two coupled oscillator networks. Each oscillator receives inhibition from the oscillators in its own network and from the oscillators in the other networks. In addition, each oscillator receives uncorrelated noisy input.

Our key finding is that in interconnected networks (Fig. 1) noise can synchronize the collective oscillations (population rhythms) that are generated by each of the oscillator networks. Importantly, the noise induces this synchronization even though it is uncorrelated between different oscillators and networks. This is in sharp contrast to the well-studied stochastic synchronization where the synchronization of different oscillators is due to the correlations in their input^37–39^. In that case the synchronization essentially reflects the transfer of correlations from the input to the output^40–42^.

We identify the mechanism that drives the synchronization of the different rhythms as a network mechanism; it arises from the noise-driven phase heterogeneity of the oscillators within each network, which allows the inter-network coupling to suppress the activity of a variable fraction of oscillators in a given cycle of the rhythm. Thus, desynchronization within each network enhances the mutual synchronizability of the networks.

This type of synchronization arises quite generally. We demonstrate it for networks comprised of various types of synaptically pulse-coupled neuronal oscillators (integrate-fire neurons, Morris-Lecar neurons of Type 1 and of Type 2) as well as for interconnected networks of relaxation oscillators that are coupled by rapid diffusion. Moreover, we find this synchronization in networks with all-to-all connectivity and in random networks.

An early account of some of these results has been previously presented^43^.

## Results

We investigate the interaction of population rhythms in interconnected networks of synaptically coupled integrate-fire (IF) neurons, of synaptically coupled Morris-Lecar neurons, and of diffusively coupled relaxation oscillators. The network connectivities are taken to be either all-to-all or random, with an effective coupling strength that is stronger within each network than across networks. In all of the cases the individual oscillators (neurons) receive noisy inputs whose means are the same within each network, but differ across networks. Importantly, the noisy inputs to different neurons belonging to the same or to different networks are uncorrelated. Details of the models can be found in the Methods section.

### Noise-Induced Synchronization of IF-Networks

To illustrate the synchronization of populations by uncorrelated noise we first show results for a large number (${\mathscr{N}}=100$) of interconnected networks of integrate-fire neurons. While for brain rhythms the interaction of only a few rhythms is expected to be particularly relevant, key elements of the synchronization can be visualized and characterized better using many networks, because they allow not only the definition of an order parameter *r*_*local*_ for the within-network synchrony, but also of a global order parameter *r*_*global*_ for the synchrony across networks (cf. Eqs (14 and 15)).

**Figure 2:**
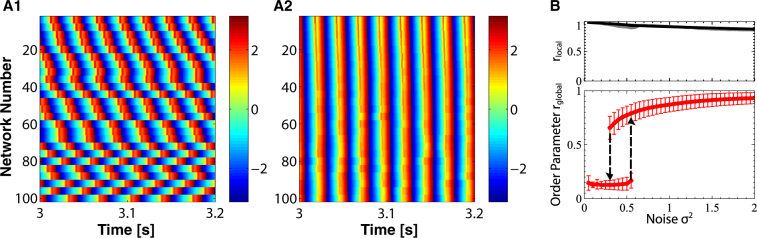
Increasing the uncorrelated noise in the inputs to the individual neurons synchronizes the population rhythms of interconnected networks of IF-neurons. (**A**) Temporal evolution of the phases *ϕ*^(*α*)^(*t*) of the population rhythms ${\bar{V}}^{(\alpha )}(t)$ of the ${\mathscr{N}}=100$ networks. (**A1**) For weak noise (*σ*^2^ = 0.04 s^−1^) the rhythms are not synchronized; shown is *ϕ*^(*α*)^(*t*) for $\alpha =4,8,12,\ldots 100$. (**A2**) Strong noise (*σ*^2^ = 2 s^−1^) synchronizes the rhythms. (**B**) The time-averaged order parameter *r*_*global*_ of the interconnected networks (lower panel) increases hysteretically with increasing strength of the uncorrelated noise (error bars denote standard deviation). The order parameters *r*^(*α*)^ of the individual networks (upper panel) decrease with noise, time-averaged *r*^(1)^ and *r*^(100)^ are shown. Parameters: *γ*_0_ = 0.0065, *g*_*syn*_ = 0.021, *μ* = 200 s^−1^, *τ* = 20 ms, *τ*_1_ = 4 ms, *τ*_2_ = 5 ms, *τ*_*d*_ = 2 ms, *V*_*rest*_ = −55 mV, *V*_*θ*_ = −45 mV, *V*_*r*_ = −65 mV, *V*_*rev*_ = −85 mV, ${\rho }^{(\alpha )}=1-0.25\,\frac{\alpha }{{\mathscr{N}}}$. The parameters *γ*_0_ and *g*_*syn*_ have been scaled so that the overall conductances of the connections within and across the networks correspond to those in the 2-network case discussed below (Fig. 3).

Thus, each row in Figs. 2A1,2 shows the collective oscillation of one of the networks. The input to each neuron consists of an independent Poisson spike train. The mean spike rate of these spike trains and with it the natural frequency of each neuron is equal for all neurons in a network but decreases with increasing index *α* of the network (for clarity only 25 networks are shown). We characterize the noise in each spike train by the variance *σ*^2^ of its spike rate. Each neuron in network *α* receives strong inhibition from all *N*_*α*_ = 100 neurons in the same network and weaker inhibition from all neurons in the other networks. Due to the strong within-network inhibition the neurons within each network synchronize, resulting in a collective oscillation (population rhythm) that corresponds to an interneuronal network *γ*-rhythm (ING)^21,44–46^. We characterize the rhythm in network *α* via the mean ${\bar{V}}^{(\alpha )}$ of the voltage *V*_*i*_ across all neurons *i* of network *α*, using it as a proxy for the local field potential (LFP) of network *α* that would typically be measured experimentally. To characterize the degree to which the different LFPs ${\bar{V}}^{(\alpha )}$ are synchronized we use the temporal average of the order parameter *r*_*global*_(*t*), which is based on the analytic signal of ${\bar{V}}^{(\alpha )}$ (cf. Methods). Analogously, we use a local order parameter ${r}_{local}^{(\alpha )}(t)$ to quantify the synchronization within network *α*, which is based on the analytic signal of the individual oscillators in network *α* (Eqs (14 and 15)).

Without or with very weak noise *σ*^2^ the LFPs ${\bar{V}}^{(\alpha )}$ of the different networks do not synchronize (Fig. 2A1); instead they oscillate at different frequencies reflecting the different mean inputs *ρ*^(*α*)^ that the neurons in the different networks receive, ${\rho }^{(\alpha )}={\rho }_{max}-\frac{\alpha }{{\mathscr{N}}}\,({\rho }_{max}-{\rho }_{min})$ with *ρ*_*max*_ = 1 and *ρ*_*min*_ = 0.75. Correspondingly, the global order parameter *r*_*global*_ is small (Fig. 2B). However, as the noise is increased above a critical value ${\sigma }_{c+}^{2}=0.44\,{{\rm{s}}}^{-1}$ the system undergoes a discontinuous transition reflected in a large jump of the order parameter *r*_*global*_. The LFPs of most networks are now synchronized (Fig. 2A2). If the noise amplitude is now reduced adiabatically, synchronization across the networks persists up to a lower value ${\sigma }_{c-}^{2}=0.24\,{{\rm{s}}}^{-1}$, revealing hysteresis. Why does this network of oscillator networks become more coherent when it is exposed to stronger uncorrelated noise?

To identify the mechanism by which uncorrelated noise can synchronize interconnected networks of oscillators we reduce the complexity of the system in two steps. We first consider two coupled networks and then the even simpler case in which network 2 is exposed to strictly periodic inhibition. We present here the results for 2 networks with all-to-all coupling. Very similar results are obtained with sparse, random connectivity in which the in-degree (but not the out-degree) is fixed (cf. Supplementary Fig. S2).

**Figure 3:**
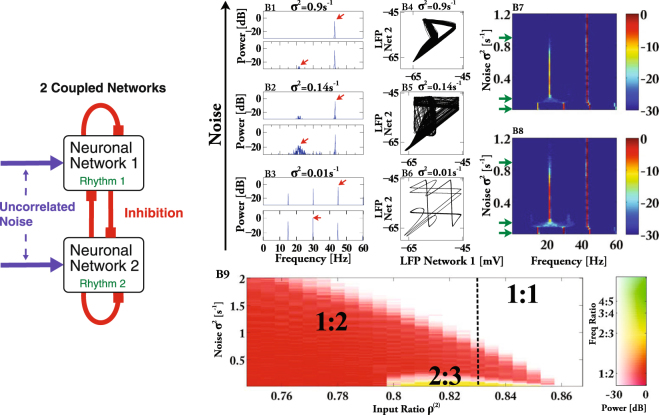
Uncorrelated noise synchronizes population rhythms of two coupled networks. (**A**) Sketch of the two coupled networks. (**B**,**B1**–**3**) Fourier spectra of LFP ${\bar{V}}^{\mathrm{(1)}}$ (upper panel) and LFP ${\bar{V}}^{\mathrm{(2)}}$ (lower panel) of network 1 and 2, respectively, on a logarithmic scale for *ρ*^(2)^ = 0.83 and different noise strengths (*σ*^2^ = 0.9 s^−1^, *σ*^2^ = 0.14 s^−1^, *σ*^2^ = 0.01 s^−1^). (**B4**–**6**) attractors for the corresponding values of *σ*^2^. (**B7**) Spectral power for network 1 as a function of noise and frequency for *ρ*^(2)^ = 0.83. Green arrows indicate noise values in (**B1**–**3**). (**B8**) as (**B7**) for network 2. (**B9**) Phase diagram. Color hue and saturation indicate frequency ratio and logarithmic power ratio of the characteristic Fourier modes (marked with red arrows in panels (**B2,3**)), respectively. Synchronization is obtained in the white region labeled 1:1. Parameters: *N*_*α*_ = 500, *τ* = 20 ms, *τ*_1_ = 4 ms, *τ*_2_ = 5 ms, *τ*_*d*_ = 2 ms, *V*_*rest*_ = −55 mV, *V*_*θ*_ = −45 mV, *V*_*r*_ = −65 mV, *V*_*rev*_ = −85 mV, *g*_*syn*_ = 0.0042, *γ*_0_ = 0.64, *μ* = 200 s^−1^.

Consistent with our results for many interconnected networks (Fig. 2), increasing the strength *σ* of the uncorrelated noise - at fixed coupling strength - can enhance the synchrony of the two rhythms of the two coupled networks (Fig. 3). For vanishing and very weak noise the two rhythms exhibit 2:3 phase-locking, as is apparent from the attractor, here represented in terms of the LFPs ${\bar{V}}^{(\alpha )}$ of the two networks (Fig. 3B6 for *ρ*^(2)^ = 0.83), and the corresponding LFP-spectra (Fig. 3B3). In this regime the two networks behave like two individual oscillators. For somewhat larger noise the attractor becomes smeared out and the spectra suggest a transition to noisy 1:2 phase locking (Fig. 3B2,5). Strikingly, a further increase in noise strength ‘cleans up’ the attractor (Fig. 3B4). This is reflected in a strong reduction of the low-frequency components of the Fourier spectra, which for sufficiently strong noise reveal 1:1 phase locking, i.e. synchronization of the two rhythms (Fig. 3B1). More detailed simulations show that the spectral peak characterizing the 1:2 phase locking decreases to very small values smoothly (Fig. 3B7,8; For clarity the frequency resolution has been reduced in Fig. 3B7,8; for each bin the maximal value of the power in that bin is shown), indicating that synchrony is reached by undergoing a continuous (super-critical) period-doubling bifurcation in reverse (see Section Period Doubling below).

The frequency ratio of the two rhythms in the absence of noise depends on the ratio *ρ*^(2)^ of the mean inputs *μ*^(*α*)^ of the two networks. Delineating the different phase-locked states as a function of *ρ*^(2)^ and of the noise strength *σ* leads to domains akin to Arnold tongues in which, strikingly, the coupling strength is replaced by the noise strength as the second control parameter. In Fig. 3B9 the color hue indicates the ratio *ω*_2_:*ω*_1_, where *ω*_1_ is the frequency of the dominant spectral peak of network 1 and *ω*_2_ is the frequency of the dominant peak of network 2 that satisfies *ω*_2_ < *ω*_1_ (arrows in Fig. 3B2,3). The saturation of the color gives the corresponding ratio *A*^2^(*ω*_2_)/*A*^2^(*ω*_1_) of the amplitudes of the peaks on a logarithmic scale. Thus, over quite some range in the input ratio *ρ*^(2)^ noise induces synchrony (white region labeled 1:1) via a continuous period-doubling bifurcation, as signified by the fading-away of *A*^2^(*ω*_2_)/*A*^2^(*ω*_1_) with *ω*_2_:*ω*_1_ = 1:2. Depending on *ρ*^(2)^, the 1:2 phase-locked state can arise directly at vanishing noise or via a transition from the 2:3 tongue.

**Figure 4:**
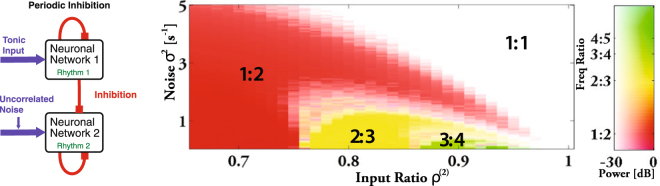
Noise increases the frequency range of entrainment of the periodically inhibited rhythm. (**A**) Periodically forced single network 2. (**B**) Phase diagram for a single network with periodic inhibition. Each neuron in the network receives uncorrelated noise, which synchronizes the rhythm with the forcing in the white region marked 1:1. Parameters and colors as in Fig. 3B9.

Analogous to the case of the network of networks (Fig. 2), where in the synchronized state the overall frequency of the system is close to that of the fastest network (Supplementary Fig. S1), the two networks synchronize at the frequency of the faster network. This suggests that the mechanism does not require the mutual interaction of the two networks, but can also operate in a single network that is exposed to strictly periodic inhibition. We implement periodic forcing by giving tonic input to network 1 and removing the inhibition it receives from network 2 (Fig. 4A). Network 1 therefore acts as a pacemaker for network 2, a situation that is, for instance, relevant for circadian rhythms with the dark-light schedule functioning as pacemaker. Indeed, depending on *ρ*^(2)^, as the noise is increased the periodically inhibited single network 2 undergoes transitions between different phase-locked states and eventually reaches the synchronized 1:1-state via a continuous period-doubling bifurcation (Fig. 4B). In parallel, the correlation between the rhythm in network 2 and the periodic forcing increases (Supplementary Fig. S6A). Figure 4B shows also that the frequency range over which the rhythm can be entrained 1:1 by the external forcing increases substantially with noise. Thus, noise enhances the synchronizability of the rhythm.

### Within-Network Desynchronization Enhances Synchronizability of Networks

**Figure 5:**
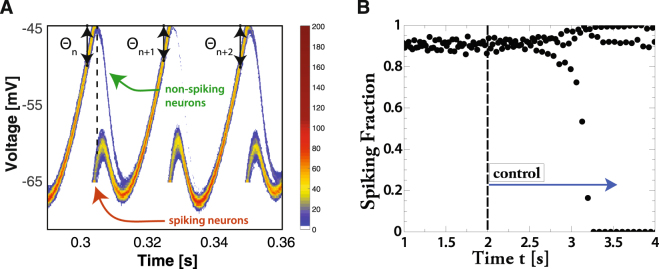
(**A**) Noise increases the synchronizability of rhythms by allowing a variable number of neurons to spike. Time-dependence of the voltage distribution function of the oscillators in the periodically forced network 2 with color indicating the number of neurons in bins of size 0.2 mV. Also shown is the lag *θ*_*n*_ in each cycle. The red (green) arrow marks spiking (non-spiking) neurons. Parameters as in Fig. 4 except for *ρ*^(2)^ = 1.02, *γ*_0_ = 0.81, *σ*^2^ = 0.3 s^−1^. (**B**) Synchrony is lost and replaced by a 3-cycle when the self-inhibition is rendered independent of the spiking fraction *f*_*spiking*_ (*t*) via the control defined by equation (1) at t = 2 s. Parameters as in Fig. 4 except for *ρ*^(2)^ = 0.975, *σ*^2^ = 2 s^−1^.

Insight into the synchronization mechanism is gained from the temporal evolution of the voltage distribution function of the neurons in the periodically forced network 2 (Fig. 5, cf. Fig. 4). The tonically driven neurons in network 1, which provides the periodic forcing, reach the threshold *V*_*θ*_ = −45 mV at the times marked by double-arrowed lines; they spike and their voltage is reset to *V*_*r*_ = −65 mV. At the dashed line the ensuing delayed inhibition reaches network 2. Even though all neurons within network 2 receive the same mean external input, the uncorrelated noise in that input reduces their correlation (Supplementary Fig. S6B) and induces a spread in their voltage. While that spread is not large in Fig. 5, it is sufficient to split the neuron population into two groups: a faster group that has already spiked when the inhibition arrives and a slower, lagging group that is kept from spiking by the strong inhibition. In contrast to the instantaneous voltage reset to *V*_*r*_ associated with spiking (red arrow), the voltage of the slower group of neurons decreases smoothly (green arrow). Eventually, due to the strong inhibition originating from the spiking neurons in network 2 the two groups of neurons in network 2 merge again before the cycle resumes.

Importantly, the self-inhibition of network 2 delays the time to its next spiking volley. Consequently, when the inhibition from network 1 keeps the lagging neurons in network 2 from spiking, the total self-inhibition within network 2 is reduced, speeding up its rhythm in that cycle. If network 2 catches up, more of its neurons escape the inhibition by network 1 and spike (cf. cycle starting at *θ*_*n*+1_ in Fig. 5), increasing self-inhibition of network 2 and slowing down its rhythm. Thus, even though the inhibition from network 1 briefly delays each neuron in network 2, overall it speeds up the rhythm of network 2 in a phase-dependent fashion. This provides a stabilizing feedback and allows the network to adjust its population frequency over a wide range.

To confirm this synchronization mechanism we remove the stabilizing feedback by adjusting in each cycle the strength *g*_*syn*_ of the self-inhibition in network 2 to compensate for the variable fraction *f*_*spiking*_(*t*) of spiking neurons,

1 $${g}_{syn}\to {g}_{syn}\frac{{\bar{f}}_{spiking}}{{f}_{spiking}(t)},$$

where ${\bar{f}}_{spiking}$ is the time average of *f*_*spiking*_ (*t*) before the control is turned on. Indeed, synchronization is lost when this control is applied, as is apparent from the resulting strongly varying spiking fraction (Fig. 5B).

**Figure 6:**
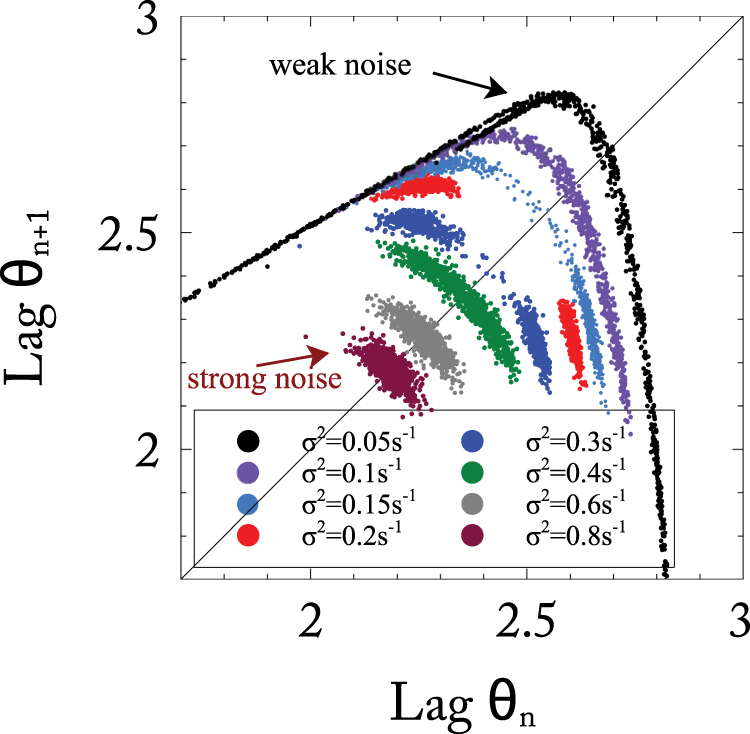
The iterated map for the lag *θ*_*n*_ of a periodically forced IF-network exhibits a period-doubling bifurcation (cf. Fig. 5). Different colors show *θ*_*n*_-sequence for one initial condition for different values of the noise *σ*^2^. With increasing *σ*^2^ the dynamics go from disordered to a noisy 2-cycle to a noisy fixed point. Parameters as in Fig. 4 except for *N*_*α*_ = 5,000, *ρ*^(2)^ = 1.03, *γ*_0_ = 0.81, *g*_*syn*_ = 4.2 × 10^−5^.

### Period-Doubling

The phase diagrams shown in Figs 3 and 4 suggest that in this parameter regime synchronization is reached via a continuous period-doubling bifurcation. To confirm this explicitly for the single network with periodic forcing (Fig. 4), we extract from the simulations an iterated map for the lag ${\theta }_{n}\equiv {V}_{\theta }-{\bar{V}}^{\mathrm{(2)}}({t}_{n})$ where ${\bar{V}}^{\mathrm{(2)}}$ is the LFP of network 2 and *t*_*n*_ is the time when neurons in network 1 spike in the *n*^*th*^ cycle; the *θ*_*n*_ are marked by double arrows in Fig. 5. For small noise (*σ*^2^ = 0.05 s^−1^) the iterates of *θ*_*n*_ trace out an almost continuous noisy attractor, corresponding to irregular dynamics (Fig. 6). When the noise is increased to *σ*^2^ = 0.2 s^−1^, this attractor changes to two domains, corresponding to a noisy 2-cycle. With a further increase in the noise (*σ*^2^ = 0.6 s^−1^) the two domains merge to a single, noisy fixed point that corresponds to the synchronized state. Conversely, when the noise is decreased, the synchronous state becomes unstable via a period-doubling bifurcation. In view of Fig. 5 this instability can be understood intuitively by noting that for weak noise the voltage distribution of the neurons is narrow and even small changes in the timing of network 2 strongly affect its fraction *f*_*spiking*_ of spiking neurons, resulting in a large gain in the feedback via self-inhibition. If that gain is too large, i.e. if the voltage distribution is too narrow, the feedback destabilizes the fixed point via a period-doubling bifurcation. This central role of the noise-induced heterogeneity in the voltage can be condensed into a simple, heuristic map model (cf. Supplementary Information, Fig. S3).

**Figure 7:**
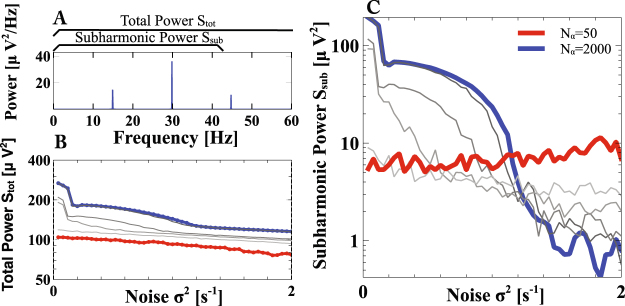
Synchronization by noise requires a minimal network size *N*_*α*_. (**A**) Spectrum of the LFP of network 2 indicating the frequency range included in the total power *S*_*tot*_ and the subharmonic power *S*_*sub*_. (**B**) *S*_*tot*_ depends only moderately on noise strength and network size (network sizes as given in the legends of panel (C)). (**C**) Only for sufficiently large networks the subhamornic spectral power *S*_*sub*_ decreases strongly with increasing noise strength indicating synchronization (note the logarithmic scale). Network sizes *N*_*α*_: 50 (red), 100, 200, 500, 1,000, 2,000 (blue). Other parameters as in Fig. 4.

Thus, desynchronization of the neurons within a network can enhance the synchronizability of the collective oscillation of a network with externally applied periodic inhibition or with the inhibition provided by rhythms in other networks.

### Minimal Network Size for Synchronization

The synchronization mechanism identified in Fig. 5 is specific to population rhythms of networks rather than oscillations of individual neurons, since it requires the number of spiking neurons and the associated inhibition within the network to decrease with increasing lag of the network. The discreteness of the network size suggests that in small networks the inhibition will be too coarsely quantized to stabilize synchrony. This is indeed the case. While the total spectral power *S*_*tot*_ of the LFP of network 2, which characterizes the strength of the rhythm itself, depends only weakly on noise and network size (Fig. 7B), the subharmonic spectral power *S*_*sub*_ of that LFP, which includes only the frequencies below the dominant frequency, decreases substantially with increasing noise in large networks, confirming the synchronization (note the logarithmic scale in Fig. 7C). As the network size is reduced, however, this decrease in *S*_*sub*_ with noise becomes smaller and for networks of size *N*_*α*_ < 100 the subharmonic power is quite independent of the noise, indicating that noise does not synchronize the rhythms in such small networks.

**Figure 8:**
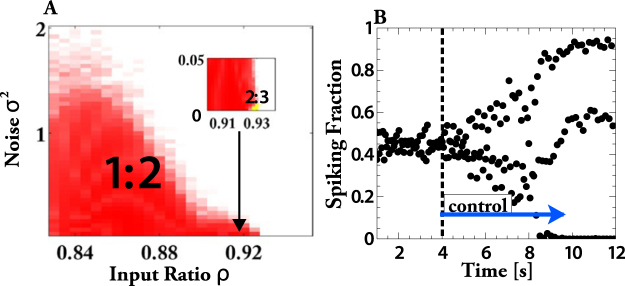
Noise synchronizes also coupled networks of type-2 Morris-Lecar neurons. (**A**) Phase diagram showing synchronization of two networks with increasing noise (inset shows blow-up). Colors as in Fig. 4. For parameters see Methods. (**B**) Loss of synchrony after turning on the control (equation (1)) for *ρ*^(2)^ = 0.84, *σ*^2^ = 2.5.

### Networks of Morris-Lecar Neurons

The key elements of the synchronization are the heterogeneity of spike timing and the dependence of the frequency of the rhythm on the strength of the inhibition. This suggests that synchronization by uncorrelated noise should be found more generally in network rhythms that arise from inhibition. To test this we replace the IF-neurons with type-2 Morris-Lecar neurons (Fig. 8). They have a very different phase-resetting curve than the IF-neurons, i.e. they respond very differently to weak *δ*-spike inputs. Consequently, for weak coupling individual Morris-Lecar neurons have very different synchronization properties than individual IF-neurons^47^. As for the IF-neurons, the interaction between the Morris-Lecar neurons is taken to be via stereotpical inhibitory synaptic pulses that are triggered when *V* reaches a threshold of *V*_*θ*_ = 10 *mV* and are described by equations (3 and 4). In these simulations we use a random, sparse connectivity in which each of the *N*_*α*_ oscillators in each network has a fixed number ${\epsilon }_{1}{N}_{\alpha }$ of randomly chosen incoming connections from the oscillators within the same network and a lower, fixed number ${\epsilon }_{2}{N}_{\alpha }$ of connections from the other network. Thus, the in-degree, but not the out-degree, of each oscillator is fixed.

**Figure 9:**
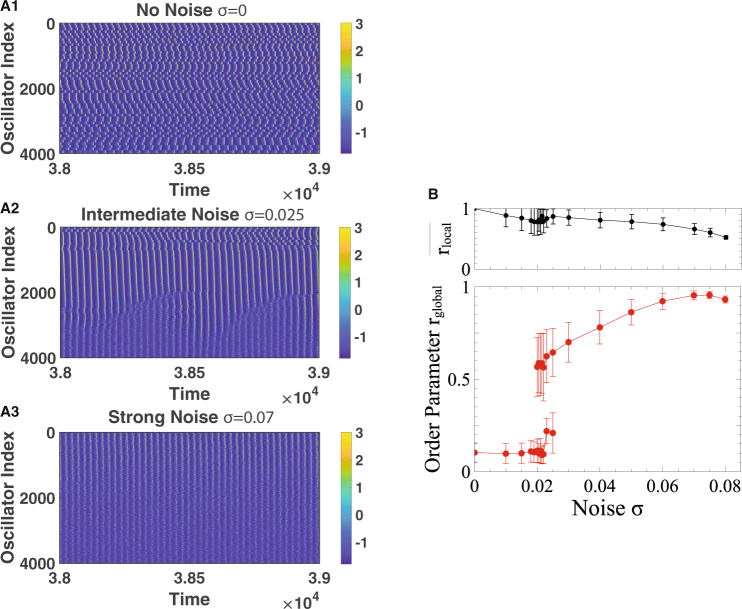
Noise synchronizes interconnected networks of relaxation oscillators. (**A**) Space-time diagram of ${x}_{i}^{(\alpha )}(t)$ for 50 of the ${\mathscr{N}}=200$ networks with *N*_*α*_ = 80 oscillators each. Networks are not synchronized for vanishing noise, *σ* = 0 (**A1**). Partial, oscillatory synchronization for *σ* = 0.025 (**A2**). Almost complete synchronization for *σ* = 0.07 (**A3**). (**B**) Lower panel: temporal mean and standard deviation of the global order parameter *r*_*global*_ showing a discontinuous transition to an ordered regime as the uncorrelated noise is increased. Upper panel: mean local order parameter ${\bar{r}}_{local}$ decreases with noise.

Again, uncorrelated noise synchronizes the population rhythms (Fig. 8A). For the parameters in Fig. 8A rhythms other than 1:2 and 1:1 arise only for very small noise (inset of Fig. 8A). For other parameter values of the inhibition and for Morris-Lecar neurons of type 1 other phase-locked states can arise for weak noise. This is shown in Supplementary Figs S4 and S5, which also demonstrate the robustness of the synchronization. As found for IF-neurons, turning on the feedback control (equation (1)), ${g}_{syn}\to {g}_{syn}{\bar{f}}_{spiking}/{f}_{spiking}(t)$, destroys the synchronization (Fig. 8B), confirming the same synchronization mechanism.

### Diffusively Coupled Networks of Relaxation Oscillators

In the neuronal models the interaction between the oscillators is via synaptic inhibition that is triggered by the oscillator and that has a stereotypical, possibly delayed waveform, the amplitude and duration of which are independent of the waveform and frequency of the oscillator. In particular, an oscillation that barely reaches threshold provides full inhibition, whereas a slightly smaller oscillation generates no inhibition at all. To address the question whether uncorrelated noise can synchronize population rhythms beyond this neuronal context we investigate interconnected networks of relaxation oscillators that are coupled via an additional field that is driven directly by one of the oscillator variables in a graded fashion (equations (11)–(13)). The coupling therefore reflects the waveform, amplitude, and duration of the ongoing oscillation and is similar to that used in various models of quorum sensing^48–50^.

**Figure 10:**
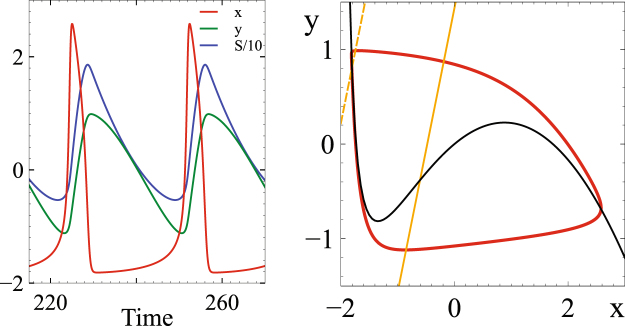
In the relaxation oscillator (equations (11,12,13)) the coupling field *S* reflects the oscillation wave form and affects the stability of the fixed point. (**A**) Temporal evolution of *x*, *y*, and *S* for the relaxation oscillator and (**B**) corresponding projection onto the (*x*, *y*)-phase plane. Black: *x*-nullcline, gold: *y*-nullclines corresponding to the minimal (solid) and the maximal (dashed) value of *S* along the limit cycle. Parameters: $\epsilon =0.05$, *β* = 3, *μ* = 0.2, *λ* = 0.05, *ν* = 2, *κ*_*local*_ = 5, *κ*_*global*_ = 0.15, ${x}_{0}^{\mathrm{(1)}}=-\,2$, ${x}_{0}^{\mathrm{(2)}}=-\,1.2$, *γ* = 2.5, *σ* = 0.

To assess the synchronization among a large number of interconnected networks we use a weighted overall order parameter *r*_*global*_(*t*) that corrects for the non-uniform evolution of the phase, when it is based on the analytic signal of the relaxation oscillator (cf. Methods). Figure 10 shows results for ${\mathscr{N}}=200$ networks with *N*_*α*_ = 80 oscillators each. In contrast to Fig. 2 where the temporal evolution of the phase *ϕ*^(*α*)^(*t*) of the population rhythm is shown, Fig. 9A shows the evolution of ${x}_{i}^{(\alpha )}(t)$ of individual oscillators. For clarity only the oscillators in every fourth network are displayed. To vary the natural frequency of the oscillators in different networks we set ${\gamma }_{i}^{(\alpha )}=1.9+1.2(\alpha -\mathrm{1)}/({\mathscr{N}}-\mathrm{1)}$, $\alpha =1\ldots {\mathscr{N}}$. The strong coupling within each of the interconnected networks synchronizes the oscillators within each network, but for vanishing noise these collective oscillations are not synchronized or phase-locked (Fig. 9A1). Thus, the average ${\bar{r}}_{local}$ of the order parameters ${r}_{local}^{(\alpha )}$ of the individual networks is high, but the time-averaged global order parameter *r*_*global*_ is low (Fig. 9B). However, similar to the case of the IF-networks (Fig. 2), as the uncorrelated noise is increased - at fixed coupling strength - the system undergoes a discontinuous transition to a state in which the rhythms of most networks are synchronized, reflected in a jump in *r*_*global*_ (Fig. 9A2,B).

Close to the transition point the system intermittently jumps between the ordered and the disordered branch; for this system size no true hysteresis is obtained. In this regime the individual order parameters ${r}_{local}^{(\alpha )}$ are much lower for the networks with low natural frequency than for the faster networks, with a sharp transition between them (Fig. 9A2). As the noise is increased further, the global order parameter rises further, while the average ${\bar{r}}_{local}$ of the individual order parameters decreases (Fig. 9A3,B). In the IF-networks the slower networks can keep up with the faster ones because their neurons spike only in fewer cycles, i.e. their spiking fraction is reduced. Similarly, the relaxation oscillators in the slower networks less often reach the right branch of the *x*-nullcline (cf. Fig. 10) than those in the faster networks, resulting in a smaller production of the rapidly diffusing substance that provides the coupling between the oscillators. This speeds up the slower networks. For yet stronger noise, not only the individual order parameteres ${r}_{local}^{(\alpha )}$ decrease, but also the global order parameter *r*_*global*_.

Interestingly, for 0.019 ≤ *σ* ≤ 0.025 the global order parameter *r*_*global*_ exhibits slow oscillations in the ordered state, which are reflected in its large standard deviation. They reflect an ‘invasion’ of the stronger disorder of the slower networks into the faster networks and a subsequent sudden retraction of this front (Fig. 9A2). Since the coupling between the networks is all-to-all, this invasion indicates that the slower networks are more susceptible to perturbations than the faster ones. The oscillations become weaker as the noise is increased. A study of these interesting oscillations is beyond the scope of this paper.

## Discussion

We have considered interconnected networks of oscillators for strong coupling of the oscillators within and across networks. This regime is beyond the weak-coupling limit and does not allow a reduction to a phase description within the framework of Kuramoto models. We have focussed on the collective oscillations (population rhythms) that emerge from the synchronization of the oscillators within each of the networks and have addressed the question to what extent these rhythms can synchronize with each other or to an external periodic pace-maker. Strikingly, we have found that uncorrelated noise can substantially enhance this synchronization.

As a key component of the underlying mechanism we have identified that the strong inter-network coupling - combined with the noise - can render the dynamics within each network highly heterogeneous with a variable subset of oscillators skipping cycles in an irregular fashion. More specifically, the synchronization of the rhythms arises from the following core aspects of the systems:

1. The synchronization of oscillators within each network is quite robust. To ensure this robustness in the neuronal models we included an explicit delay in the interaction to avoid that the neuronal populations of the uncoupled networks form clusters rather than being fully synchronized^51^.
2. The frequency of the rhythm of each network decreases with increasing coupling between the oscillators within the network. This is a characteristic feature of the ubiquitous neuronal *γ*-rhythm generated by the ING- or PING mechanism^22^.
3. The strength of the interaction between the oscillators depends on their oscillation amplitude. This allows the overall coupling within a network and with it the frequency of the rhythm to depend on the degree to which the oscillators participate in the collective oscillation. This is naturally the case in neuronal systems with chemical synapses where the output of a neuron depends on whether the neuron spikes or not. In cellular oscillators, e.g. genetic oscillators, such a coupling is likely to arise if the production of the substance that provides the communication between the cells depends on one of the oscillating variables^49,50^.
4. The inter-network coupling is sufficiently strong and acts on a time scale that allows to impact oscillators differently depending on whether they are leading or lagging the collective oscillation. In the models discussed here the inter-network coupling suppresses the oscillations of the lagging oscillators, which modifies the frequency of the rhythm they are participating in. This feedback enhances the stability of the synchronized state.

Noise is essential for the synchronization, since it spreads out the phases of the oscillators, which then allows the inter-network coupling to suppress the trailing but not the leading oscillations. In essence, the enhanced synchronizability of these collective oscillations emerges from the noise-induced desynchronization within each network. Importantly, the synchronization of different population rhythms does therefore not imply the synchronization of the oscillators within a network or across networks. In fact, with increasing noise the within-network correlations decrease.

Since the within-network desynchronization plays a key role in the synchronization mechanism, our analysis suggests that population rhythms could also be synchronized by heterogeneity in the natural frequencies of the oscillators within each network.

Since the external inhibition acting on each network modifies the self-inhibition of that network and with it its frequency, the synchronization mechanism has some similarity with that proposed in for the flash synchronization in certain species of fireflies^52^. However, there the external flash leads to a slow and persistent adaptation of the intrinsic frequency of the fireflie’s rhythm, while in the systems discussed here the frequency change is quite fast and - at least for very strong self-inhibition - it does not persist very long. This may be different for weaker self-inhibition, for which the synchronization transition does not always involve a period-doubling bifurcation (cf. Supplementary Fig. S4A).

Most of the results presented here are formulated in terms of neuronal systems, motivated by the wide-spread appearance of *γ*-rhythms in the brain and by the widely investigated hypothesis that coherence of *γ*-rhythms in different brain areas is an important element of information transmission between these areas^10–16^. The synchronization of different *γ*-rhythms can also be relevant in intertwined subnetworks of a single, modularly structured brain area^32–36^. In this context, our results point to a possible new constructive role of noise in the communication between different brain areas and the information processing within a single area.

It should be noted that most, but not all^46^, neuronal networks of the brain that exhibit *γ*-rhythms comprise inhibitory as well as excitatory neurons. Depending on the balance between excitatory and inhibitory coupling and on the associated time scales the rhythms generated by these networks can be closer to an ING-rhythm or a PING-rhythm (pyramidal interneuron network gamma)^19^. A general analysis of the effect of noise on the interaction of rhythms in two excitatory-inhibitory (EI) networks is very complex, since it involves 4 different types of connections (II, EI, IE, EE) among the neurons of the two networks. In order to be able to isolate and elucidate the synchronization mechanism in detail, we have focused in this paper on the core property that is common to ING and PING *γ*-rhythms, which is the delay of spiking by inhibition.

The noise-induced synchronization in interconnected networks is not limited to neuronal rhythms. Our results for relaxation oscillators show that it can be relevant for rhythms more generally^53^. In fact, our model is similar to a model proposed for the interaction of genetic oscillators^49^. In such systems molecular noise can be an integral component due to the small copy number of some of the participating reactants. More specifically, the synchronization of the collective oscillation of a single network of oscillators by an external forcing (cf. Fig. 4) is, for instance, relevant in the entrainment of the rhythm generated by the biomolecular circadian oscillators in the ~20,000 cells of the suprachiasmatic nucleus by the day-night cycle^54^. Interestingly, there it has been observed that transient desynchronization of the oscillations of the individual cells accelerates the entrainment of the overall rhythm after shifts in the light schedule^55^. Whether the phase-heterogeneity of the cells plays a role similar to that described here is not clear.

In cyanobacteria the coupling between the circadian rhythms of different bacteria is extremely weak^56^. However, within each bacterium the coupling between the proteins that constitute the individual oscillating units is strong^57,58^. This raises the possibility that molecular noise may enhance the entrainment of this circadian rhythm by the day-night cycle.

The type of synchronization identified here could also be amenable to experimental investigations in chemical oscillations like the Belousov-Zhabotinsky reaction where the reaction can be localized on a large number of beads and the interaction can be supplied by feedback that exploits the light sensitivity of the reaction^59^.

The global order parameter of interconnected networks can exhibit non-trivial dynamics^60^. For the networks of relaxation oscillators we find that on the upper, ordered branch, which is reached in a discontinuous transition as the noise is increased, the order parameter exhibits persistent oscillations that are associated with the invasion and retraction of front-like structures that separate more tightly synchronized networks from less synchronized ones. The origin of these dynamics is not yet understood.

## Methods

To illustrate the generality of our results we use three different types of oscillators as the nodes of the networks. Motivated by the relevance of the interaction of population rhythms for the communication between different brain areas^9,11,13,16^, we consider two different neuronal models: the single-component, discontinuous integrate-fire model and the continuous two-component Morris-Lecar model (type 1 and type 2). In both cases the interaction between the neurons is through synapses that provide pulse-like inhibition with a stereotypical wave form that is triggered when the presynaptic neuron surpasses a threshold. In the third model we go beyond the neuronal context and use relaxation oscillators that are continuously coupled. They can be thought of as individual cells that interact via a rapidly diffusing substance, similar to quorum sensing^48–50^.

### Integrate-Fire Neurons

Each neuron *i* in network *α* is characterized by the depolarization *V*_*i*_(*t*), $i=1\ldots N$, which satisfies

2 $$\tau {\dot{V}}_{i}={V}_{rest}-{V}_{i}+R\,{I}_{i}^{(syn)}(t)+R\,{I}_{i}^{(ext)}(t\mathrm{)}.$$

To avoid cluttering the equations, we do not indicate the network *α* for each neuron in this section. Here ${I}_{i}^{(ext)}(t)$ denotes a noisy external excitatory input and ${I}_{i}^{(syn)}(t)$ the total synaptic current the neurons receives, which provides the coupling between the neurons within network *α* and across networks. The parameters *τ* and *R* are the membrane time constant and the membrane resistance, respectively. When *V*_*i*_(*t*) reaches the firing threshold *V*_*θ*_, a spike is triggered and the voltage is reset to the reset voltage *V*_*r*_. In integrate-fire neurons the oscillation frequency increases continuously from 0 when the spiking threshold is surpassed^61^.

The synaptic currents are modeled as the difference of two exponentials, triggered by spikes of presynaptic neurons *j* at times ${t}_{j}^{(k)}$,

3 $${I}_{i}^{(syn)}=\frac{{g}_{syn}}{R}({A}_{i}^{\mathrm{(2)}}-{A}_{i}^{\mathrm{(1)}})\,({V}_{rev}-{V}_{i}),$$

with

4 $${\dot{A}}_{i}^{\mathrm{(1},\mathrm{2)}}=-\,\frac{{A}_{i}^{\mathrm{(1,2)}}}{{\tau }_{\mathrm{1,2}}}+\sum _{j=1}^{N}\,\sum _{k}\,{W}_{ij}\,\delta (t-{t}_{j}^{(k)}-{\tau }_{d}).$$

Here *g*_*syn*_ denotes the dimensionless synaptic strength and *τ*_*d*_ the synaptic delay. Being conductance-based, the synaptic current depends on the post-synaptic voltage *V*_*i*_ relative to the reversal potential *V*_*rev*_, which is strongly negative for the inhibitory synapses considered here. The connectivity matrix is denoted by **W** with its non-zero elements given by *W*_*ij*_ = 1 if neuron *i* and *j* belong to the same network, while *W*_*ij*_ = *γ*_0_ < 1 if they belong to different networks.

The external input of each neuron *i* is modeled as an independent Poisson-train of *δ*-spikes at times ${t}_{ik}^{(ext)}$,

5 $${I}_{i}^{(ext)}(t)=\frac{{V}_{\theta }-{V}_{r}}{R}{\rm{\Delta }}{v}_{i}\,\tau \,\sum _{k}\,\delta (t-{t}_{ik}^{(ext)}\mathrm{)}.$$

Thus, the noisy external inputs to different neurons are uncorrelated. The dimensionless input strengths Δ*v*_*i*_ are scaled such that for Δ*v*_*i*_ = 1 a single pre-synaptic input spike is sufficient to trigger a spike in the post-synaptic neuron. The input strengths are equal for all neurons within a network, but differ between networks: Δ*v*_*i*_ = Δ*v*^(*α*)^ for neurons in network *α*. The input ratio ${\rho }^{(\alpha )}\equiv {\rm{\Delta }}{v}^{(\alpha )}/{\rm{\Delta }}{v}^{\mathrm{(1)}}$ determines the frequency ratio of the rhythms of the uncoupled networks.

Instead of the spike rates *λ*^(*α*^) of the Poisson trains and the strengths Δ*v*^(*α*)^ we use the mean input *μ*^(*α*)^ = *λ*^(*α*)^Δ*v*^(*α*)^ and its variance *σ*^2^ = *λ*^(*α*)^(Δ*v*^(*α*)^)^2^ as independent parameters. Thus, the noise strength characterized by *σ*^2^ is the same for all neurons in all networks. The spike rates used in this paper are of the order ${\mathscr{O}}\mathrm{(10,000}\,{{\rm{s}}}^{-1})$, which corresponds to each neuron in the network receiving external input from ${\mathscr{O}}\mathrm{(200)}$ neurons, each firing at a rate of ${\mathscr{O}}\mathrm{(50}\,{{\rm{s}}}^{-1})$.

### Morris-Lecar Neurons

For weak coupling the synchronization between individual neuronal oscillators depends strongly on their phase-response curve, i.e. on the change in their oscillation phase in response to a small *δ*-spike input. To go beyond integrate-fire neurons, which have a type-1 phase-response curve, we also investigate synchronization in Morris-Lecar neurons, which are of type 1 or type 2 depending on the parameter values. They are described by a voltage *V* and a gating variable *n* for the potassium conductance,

6 $$\begin{array}{rcl}{C}_{m}{\dot{V}}_{i} & = & -{g}_{l}({V}_{i}-{V}_{leak})-{g}_{Ca}\,{m}_{\infty }({V}_{i})\,\,({V}_{i}-{V}_{Ca})\\  &  & -{g}_{K}\,n({V}_{i})\,\,({V}_{i}-{V}_{K})\\  &  & +\sum _{j}\,{W}_{ij}{I}_{ij}^{(syn)}+{I}_{i}^{(ext)},\end{array}$$

7 $$\begin{array}{rcl}{\tau }_{n}({V}_{i})\,{\dot{n}}_{i} & = & {n}_{\infty }({V}_{i})-{n}_{i}.\end{array}$$

Here

8 $${m}_{\infty }(V)=\frac{1}{2}(1+\,\tanh \,\frac{V-{V}_{{\theta }_{m}}}{{V}_{{s}_{m}}}),$$

9 $${n}_{\infty }(V)=\frac{1}{2}(1+\,\tanh \,\frac{V-{V}_{{\theta }_{n}}}{{V}_{{s}_{n}}}),$$

10 $${\tau }_{n}(V)=\frac{1}{\varphi \,\cosh \,\frac{V-{V}_{{\theta }_{n}}}{2{V}_{{s}_{n}}}}.$$

As for the IF-neurons, the external current ${I}_{i}^{(ext)}$ consists of Poisson spike trains with mean *μ* and variance *σ*^2^ (cf. equation (5)) and the synaptic currents are given by equation (3).

We use here *C*_*m*_ = 20 *μ*F/cm^2^, *g*_*Ca*_ = 4 ms/cm^2^, *g*_*K*_ = 8 ms/cm^2^, *g*_*L*_ = 2 ms/cm^2^, *ϕ* = 1/15 s^−1^, *V*_*Ca*_ = 120 mV, *V*_*K*_ = −80 mV, *V*_*L*_ = −60 mV, ${V}_{{\theta }_{m}}=-\,1.2\,{\rm{mV}}$, ${V}_{{s}_{m}}=18\,{\rm{mV}}$, ${V}_{{s}_{n}}=17.4\,{\rm{mV}}$. For ${V}_{{\theta }_{n}}=12\,{\rm{mV}}$ these equations describe then a type-1 neuron, while for ${V}_{{\theta }_{n}}=2\,{\rm{mV}}$ one obtains a type-2 neuron^62^. The synaptic parameters are *γ*_*syn*_ = 0.0084, *τ*_1_ = 4 ms, *τ*_2_ = 5 ms.

### Relaxation Oscillators

To go beyond the coupling by stereotypical pulses that is characteristic for neuronal systems, we consider a minimal model of relaxation oscillators that communicate through a rapidly diffusing substance *S*, reminiscent of the quorum sensing used in models for the synchronization of genetic, cellular oscillators^49,50^. Each oscillator *i* in network *α* is described by

11 $${\dot{x}}_{i}={x}_{i}-{x}_{i}^{3}-{y}_{i}({x}_{i}-{x}_{0}^{\mathrm{(1)}})+{\xi }_{xi},$$

12 $${\dot{y}}_{i}=\epsilon (\,-\,{y}_{i}+\beta {x}_{i}+{\gamma }_{i}+\mu {S}_{i})+{\xi }_{yi},$$

13 $$\begin{array}{rcl}{\dot{S}}_{i} & = & -\,\lambda {S}_{i}+\nu ({x}_{i}-{x}_{0}^{\mathrm{(2)}})\\  &  & -{\kappa }_{local}({S}_{i}-{S}_{local})-{\kappa }_{global}({S}_{i}-{S}_{global})+{\xi }_{Si}.\end{array}$$

Here *x*, *y*, and *S* can be thought of as deviations in the concentrations of the respective substances from a mean value. *S*_*local*_ is the average of *S*_*i*_ within network *α* and *S*_*global*_ is the average of *S*_*i*_ across the oscillators of all networks. The strength of the coupling within each network is given by *κ*_*local*_, whereas the global interaction among all oscillators is given by *κ*_*global*_. Thus, within and across the networks the interaction of the oscillators is all-to-all. Each component of each oscillator is driven by Gaussian white noise *ξ*_*x*,*y*,*z*_ with the same variance *σ*^2^; the noise is *δ*-correlated in time and uncorrelated across components and oscillators.

The dynamics of this model are shown in Fig. 10 for an individual oscillator. The reactant *S* responsible for the interaction is produced for ${x}_{i} > {x}_{0}^{\mathrm{(2)}}$. Thus, its amount depends on the waveform of the oscillation, particularly on its amplitude. An increase in *S* shifts the *y*-nullcline to the left, shifting the fixed point and reducing the oscillation frequency. For sufficiently large *S* the fixed point becomes stable.

### Order Parameters

In the IF-model the voltage trace *V*(*t*) does not include the action potential (spike) itself. When taking the mean across the network to obtain ${\bar{V}}^{(\alpha )}$, we therefore add a spike of size *V*_*spike*_ = 45 mV to the voltage in the time step when *V* reaches the threshold *V*_*θ*_. This enhances the diagnostics in terms of the order parameter. To characterize the degree to which the different LFPs ${\bar{V}}^{(\alpha )}$ are synchronized we use the temporal average of the order parameter *r*_*global*_ defined as

14 $${r}_{global}(t)\,{e}^{i{\psi }_{global}(t)}=\frac{1}{{\mathscr{N}}}\,\sum _{\alpha =1}^{{\mathscr{N}}}\,{e}^{i{\varphi }^{(\alpha )}(t)}.$$

Here the phase *ϕ*^(*α*)^ is the argument of the analytic signal of ${\bar{V}}^{(\alpha )}$, which is obtained via a Hilbert transform. Analogously, the local order parameter

15 $${r}_{local}^{(\alpha )}(t)\,{e}^{i{\psi }_{local}^{(\alpha )}(t)}=\frac{1}{{N}_{\alpha }}\,\sum _{k=1}^{{N}_{\alpha }}\,{e}^{i{\varphi }_{k}^{(\alpha )}(t)}$$

measures the synchronization within network *α*. Here ${\varphi }_{k}^{(\alpha )}(t)$ is the argument of the analytic signal of oscillator *k* in network *α*.

For the networks of relaxation oscillators we use an overall order parameter that is based on the argument *ϕ*^(*α*)^ of the analytic signal of the averages ${\bar{x}}^{(\alpha )}(t)\equiv \frac{1}{{N}_{\alpha }}{\sum }_{k=1}^{{N}_{\alpha }}\,{x}_{k}^{(\alpha )}(t)$ within each network *α*. The distribution function ${\mathscr{P}}({\varphi }^{(\alpha )})$, when sampled uniformly in time, turns out to be strongly non-uniform. This reflects the fact that in these relaxation oscillations the oscillators - and also the collective oscillations - spend much more time in specific parts of phase space and *ϕ*^(*α*)^ evolves quite nonlinearly in time. Because of this bias in ${\mathscr{P}}({\varphi }^{(\alpha )})$ the unweighted average ${{\mathscr{N}}}^{-1}\,{\sum }_{\alpha =1}^{{\mathscr{N}}}\,{e}^{i{\varphi }^{(\alpha )}(t)}$ across the ${\mathscr{N}}$ interconnected networks does not vanish even if the averages ${\bar{x}}^{(\alpha )}(t)$ of all of the networks are completely uncorrelated and ${\mathscr{N}}$ is large. We therefore define the order parameter via the weighted average

$${r}_{global}(t)\,{e}^{i{\psi }_{global}(t)}=\frac{1}{{\mathscr{N}}}\,\sum _{\alpha =1}^{{\mathscr{N}}}\,\frac{1}{{\mathscr{P}}({\varphi }^{(\alpha )}(t))}{e}^{i{\varphi }^{(\alpha )}(t)}.$$

We approximate ${\mathscr{P}}({\varphi }^{(\alpha )}(t))$ using a 6^*th*^-order polynomial fit to the histogram of *ϕ*^(*α*)^. With this correction the order parameter is appropriately very small when the collective oscillations are uncorrelated. In principle, the same correction should be used for the order parameters ${r}_{local}^{(\alpha )}$ of the individual networks. However, for the strong order found within the individual networks introducing the weights has only little impact. We therefore forgo this slight improvement, which requires substantial computational effort, and use the unweighted local order parameter ${r}_{local}^{(\alpha )}$ as defined in equation (15) based on the analytic signal of ${x}_{k}^{(\alpha )}(t)$.

### Data availability

The datasets generated during and/or analysed during the current study are available from the corresponding author on reasonable request.

## Electronic supplementary material

Supplementary Information
