# Supplementary material for: Synchronization by uncorrelated noise: interacting rhythms in interconnected oscillator networks
Source: Sci Rep. 2018 May 3;8:6949. doi: 10.1038/s41598-018-24670-y (PMC5934367; doi:10.1038/s41598-018-24670-y)
Supplement: Supplementary file 1 — Supplementary Information [file 41598_2018_24670_MOESM1_ESM.pdf]

SUPPLEMENTARY INFORMATION

Synchronization by uncorrelated noise: interacting rhythms in interconnected oscillator networks

John Hongyu Meng and Hermann Riecke

Spectra for Many Interconnected IF-Networks

A characteristic feature of the synchronization mechanism is that noise allows the slower network to speed up, but it does not slow down the faster network (cf. Fig. 3). This is also seen in the spectra of the large interconnected network of Fig.2. For low noise the spectrum of each network is broad (Fig.S1 top panels). However, in the ordered regime at larger noise, the frequencies of most networks has increased to the value of the fastest network.

Two IF-Networks with Random Connectivity

To demonstrate the robustness of the synchronization with respect to changes in the connectivity we also consider the impact of uncorrelated noise on two coupled IF-networks with random connectivity. As in the analysis of the Morris-Lecar networks (Fig.8) each oscillator receives $\epsilon_1 N_\alpha$ random inhibitory connections from its own network and a smaller number $\epsilon_2 N_\alpha$ of random inhibitory connections from the other network. Thus, all oscillators have the same in-degree, but not the same out-degree. We have avoided the heterogeneity that would be associated with variable in-degree in order to focus on the synchronization by temporal noise. As shown in Fig.S2, the overall behavior of these random networks is very similar to the networks with all-to-all coupling with the noise synchronizing the two networks (Fig.S2).

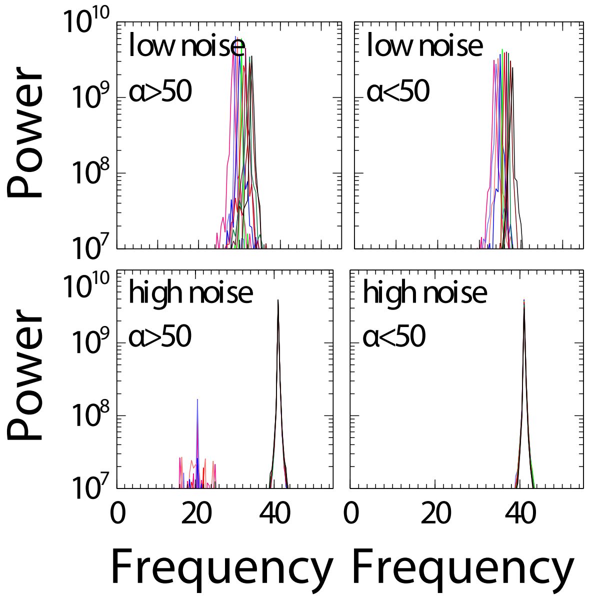

FIG. S1. Uncorrelated noise synchronizes the networks at the frequency of the fastest network. Upper panels: for low noise, $\sigma^2 = 0.04\text{s}^{-1}$, the networks are not synchronized. Fourier spectra of the LFPs $\bar{V}^{(\alpha)}$ of 25 of the 100 interconnected IF-networks shown in Fig.2 vary significantly in frequency. Lower panels: For stronger noise, $\sigma^2 = 2\text{s}^{-1}$, all networks are essentially synchronized, showing only a weak subharmonic component (note the logarithmic scale). Left (right) panels show the spectra for the networks with weaker ($\alpha > \mathcal{N}/2$) and stronger ($\alpha \leq \mathcal{N}/2$) injected current. Parameters as in Fig.2.

A Heuristic One-dimensional Map Model

The simplicity of the mechanism identified in our simulations (Fig.5) suggests that its essence may be captured in a simplified model. The temporal evolution of the voltage distribution of the neurons in network 2 shows that the voltage distribution is quite sharply peaked. Moreover, shortly before the times when the periodic inhibition arrives in network 2 the distribution is close to unimodal and can be characterized by the LFP of network 2 and its lag relative to network 1 or the periodic inhibition. This allows us to develop a phenomenological Poincare map for the lag θ.

Supplementary Figs.S4,S5 indicate that the synchronization mechanism is not very sensitive to the voltage dependence of the inhibition. For simplicity we therefore assume that the inhibition resets the voltage of an oscillator by an amount proportional to its voltage and write the evolution of a normalized mean voltage (LFP) $\bar{V}$ as

$$\dot{\bar{V}} = \rho_{map} - g_1\,\bar{V}(t)\sum_{n=-\infty}^{\infty}\delta\left(t-(n+\tau_d)\right) - g_2\,P(\bar{V}(t-\tau_d))\,\bar{V}(t), \quad (16)$$

with $\bar{V}$ being reset to $\bar{V}=0$ instantaneously when it reaches $\bar{V}=1$. The second term in equation (16) represents a periodic external forcing with strength g_1 and period 1. The third term models the self-inhibition of the network. Its strength depends on the number $P(\bar{V}(t))$ of oscillators that are at the spike threshold when the LFP has the value $\bar{V}$. The evolution of $\bar{V}(t)$ is shown in supplementary Fig.S3A.

$P(\bar{V}(t))$ reflects the voltage distribution of the oscillators, which results in a heterogeneity in the spike times of the oscillators in network 2. The simulations of the integrate-fire model indicate that this heterogeneity plays a central role (Fig.5). Instead of considering an evolution equation for the voltage distribution, for our minimal model we consider it time-independent and of the form

$$P(\bar{V}) = \begin{cases} \frac{1}{\sigma_{map}} & \bar{V}\in[0,\frac{1}{2}\sigma_{map}]\cup[1-\frac{1}{2}\sigma_{map},1) \\ 0 & \text{otherwise.}\end{cases} \quad (17)$$

Thus, for $\bar{V}\in[0,\sigma_{map}/2]$ neurons in the trailing half of the distribution are firing, while for $\bar{V}\in[1-\sigma_{map}/2,1)$ neurons in the leading half are firing.

With $n+\tau_d$ the time at which the periodic inhibition arrives in the nth-cycle and letting t_n be the time at

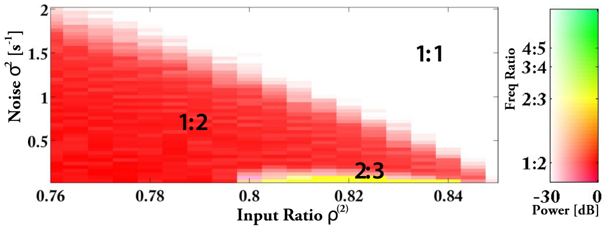

FIG. S2. Uncorrelated noise synchronizes population rhythms of two coupled IF-networks with random connectivity. Phase diagram showing transitions between different phase-locked and synchronized states as a function of noise and input ratio. Color hue and saturation indicate frequency ratio and logarithmic power ratio of the dominant Fourier modes (cf. Fig.3). Parameters: $N_\alpha = 500$, $\epsilon_1 = 0.56$, $\epsilon_2 = 0.24$, $\tau = 20\text{ms}$, $\tau_1 = 4ms$, $\tau_2 = 5\text{ms}$, $\tau_d = 2\text{ms}$, $V_{rest} = -55\text{mV}$, $V_\theta = -45\text{mV}$, $V_r = -65\text{mV}$, $V_{rev} = -85\text{mV}$, $g_{syn} = 0.015$, $\gamma_0 = 1.5$, $\mu = 200s^{-1}$.

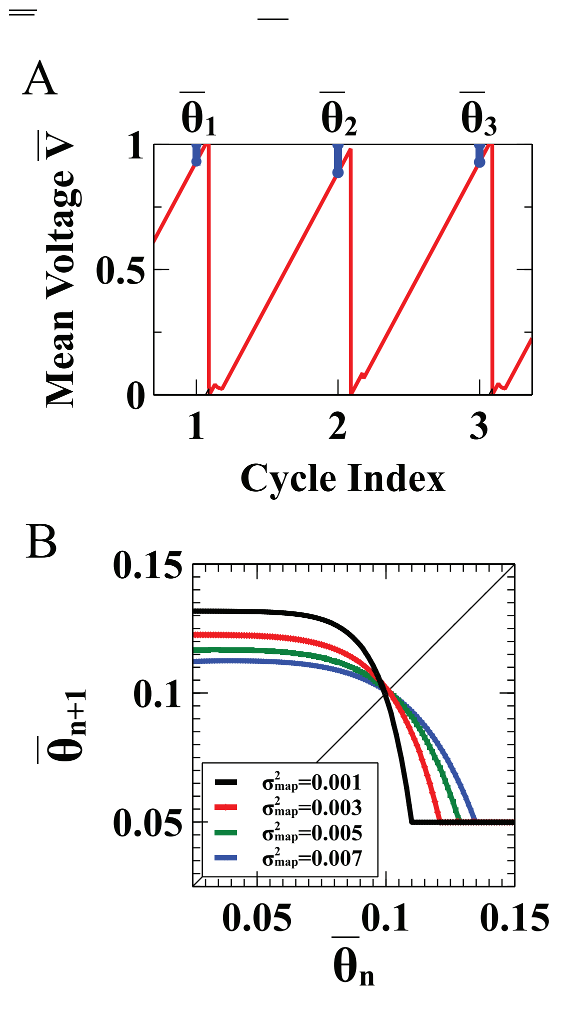

FIG. S3. The synchronization via reverse period-doubling can be captured in a heuristic map model. A) Temporal evolution of the normalized mean voltage $\bar{V}$, with lag $\bar{\theta}_n$ indicated and plotted in terms of the cycles of the periodic inhibition. B) Map given by equation (19). The fixed point becomes stable with increasing noise level σ^2_{map}.

which $\bar{V}$ reaches threshold, $\bar{V}(t_n) = 1$, we focus on the situation in which the external inhibition arrives before any of the self-inhibition sets in that is triggered by the oscillators in network 2,

$$n + \tau_d < t_n - \frac{\sigma_{map}}{2\rho_{map}} + \tau_d.$$

The external inhibition induces a reset

$$\bar{V}(n + \tau_d) \rightarrow e^{-g_1}\bar{V}(n + \tau_d).$$

For sufficiently strong coupling g_1 it keeps the trailing oscillators from spiking and from contributing to the self-inhibition. Thus, self-inhibition lasts from

$$t_n^< = t_n - \frac{\sigma_{map}}{2\rho_{map}} + \tau_d$$

to

$$t_n^> = \min\left(n + 2\tau_d, t_n + \frac{\sigma_{map}}{2\rho_{map}} + \tau_d\right).$$

During that time $\Delta t \equiv t_n^> - t_n^<$ it induces a voltage change that leads to

$$\bar{V}(t_n^>) = e^{-g_2\frac{\rho_{map}}{\sigma_{map}}\Delta t}\bar{V}(t_n^<) + \frac{\sigma_{map}}{g_2}(1 - e^{-g_2\frac{\rho_{map}}{\sigma_{map}}\Delta t}). \tag{18}$$

Combining equation (18) with the voltage evolution during the remaining time yields a Poincare map for the lag $\bar{\theta}_n \equiv 1 - \bar{V}(n)$ of network 2 relative to the periodic inhibitory input (supplementary Fig.S3B),

$$\bar{\theta}_{n+1} = F(\bar{\theta}_n). \tag{19}$$

The fixed point $\bar{\theta}_{FP} = F(\bar{\theta}_{FP})$ corresponds to a 1:1 synchronized state. Its stability depends on the slope $F'(\bar{\theta}_{FP})$. It is only stable $(|F'(\bar{\theta}_{FP})| < 1)$ for large widths σ_{map} of the distribution P, i.e. for sufficiently strong noise, and becomes unstable via a period-doubling bifurcation at $F'(\bar{\theta}_{FP}) = -1$ as the noise is reduced.

Thus, this simple map model extracts the key role of the noise-induced heterogeneity of the spike times in network 2 in the synchronization of the population rhythms found in the full network simulations (Figs.3,4,8) and gives further support for the mechanism that we extracted from our simulations (Figs.5,8).

Dependence of Synchronization on the Duration of Inhibition and the Reversal Potential

To assess the generality of the synchronization mechanism we vary key aspects of the inhibition: its temporal evolution and its reversal potential V_{rev}. To vary the effective delay and the duration of the inhibition we

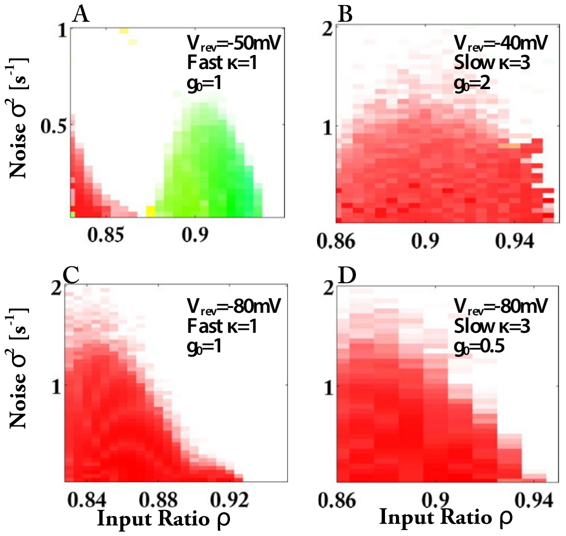

FIG. S4. Phase diagrams for networks of type-2 Morris-Lecar neurons demonstrate that increasing noise synchronizes the rhythms for fast ($\kappa = 1$) and slow inhibition ($\kappa = 3$) and over a large range in reversal potential V_{rev}. Parameters as in Fig.8 with $V_{\theta_n} = 2$mV.

rescale the rise and decay times $\tau_{1,2}$ by a common factor κ,

$$\dot{A}_i^{(1,2)} = -\frac{A_i^{(1,2)}}{\kappa\, \tau_{1,2}} + \sum_{j=1}^{N} \sum_{k} W_{ij}\, \delta\left(t - t_j^{(k)} - \tau_d\right). \quad (20)$$

For $\kappa > 1$ this shifts the decay and the peak of the inhibition to later times. The latter amounts to an increase in the effective delay. The reversal potential V_{rev} determines the dependence of the inhibition on the voltage of the cell receiving the inhibition,

$$I_i^{(syn)} = g_0\, \frac{g_{syn}}{R} \left(A_i^{(2)} - A_i^{(1)}\right) (V_{rev} - V_i)\,. \quad (21)$$

We have included a factor g_0 in Eq.(21), which indicates the change in the synaptic strength used in Figs.S4,S5 compared to the main part of the paper.

If the reversal potential is significantly below the resting potential of the cell, the conductance-based inhibition is very similar to inhibition by a fixed negative current. It shifts the voltage of the fixed point that corresponds to the non-spiking state to more negative (hyperpolarized) values. However, if the reversal potential is at the resting potential, inhibition vanishes for the non-spiking cell and the location of the fixed point is not affected. Nevertheless, synaptic input increases the conductance of the cell and functions as a shunt for any excitatory inputs, stabilizing the fixed point. With respect to the formation of a γ-rhythm within an individual network it is known that with shunting inhibition type-2 neurons tend to synchronize only poorly and the γ-rhythms themselves tend to be ffragile [64].

We find that uncorrelated noise synchronizes the rhythms over a wide range of the time scale of the inhibi-

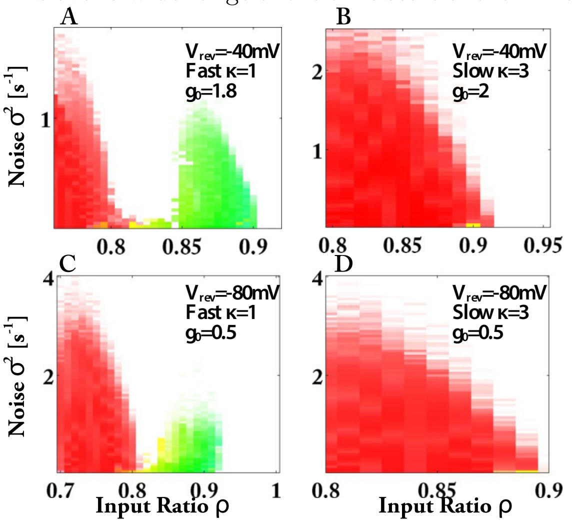

FIG. S5. Phase diagrams for networks of type-1 Morris-Lecar neurons demonstrate that increasing noise synchronizes the rhythms for fast ($\kappa = 1$) and slow inhibition ($\kappa = 3$) and over a large range in reversal potential V_{rev}. Parameters as in Fig.8 with $V_{\theta_n} = 12$mV.

tion and of the reversal potential for networks of type-1 neurons (supplementary Fig.S5) and of type-2 neurons (supplementary Fig.S4). Here we use the Morris-Lecar model for both types of neurons. Interestingly, in some cases the synchronization does not involve a period-doubling bifurcation (supplementary Figs.S4A, S5A,C). For values of the reversal potential close to the resting potential synchronization does not occur for type-2 Morris-Lecar neurons, reflecting the fragility of the γ-rhythms themselves [64].

Correlations

The synchronization of the population rhythms does not imply the synchronization of individual oscillators. To demonstrate this we measure the equal-time correlation between the voltages of the individual neurons within network 2 ($\langle V_j^{(2)}, V_k^{(2)} \rangle$) and compare it with the correlation between the LFP of network 2 and the periodic forcing ($\langle \bar{V}^{(2)}, \bar{V}^{(1)} \rangle$). For the latter we determine the phase shift between the two signals from the difference in the phases of their dominant Fourier modes and plot in supplementary Fig.S6A the correlation for that delay as a function of the noise and the input ratio $\rho^{(2)}$. Mirroring the phase diagram of the periodically forced network (Fig.4) the correlation between the LFP of network 2 and the periodic forcing increases with noise. However, the voltage correlations between the individual neurons within network 2 decrease monotonically with increasing noise strength (supplementary Fig.S6B), reflecting the decrease in the local order parameters in Fig.2B.

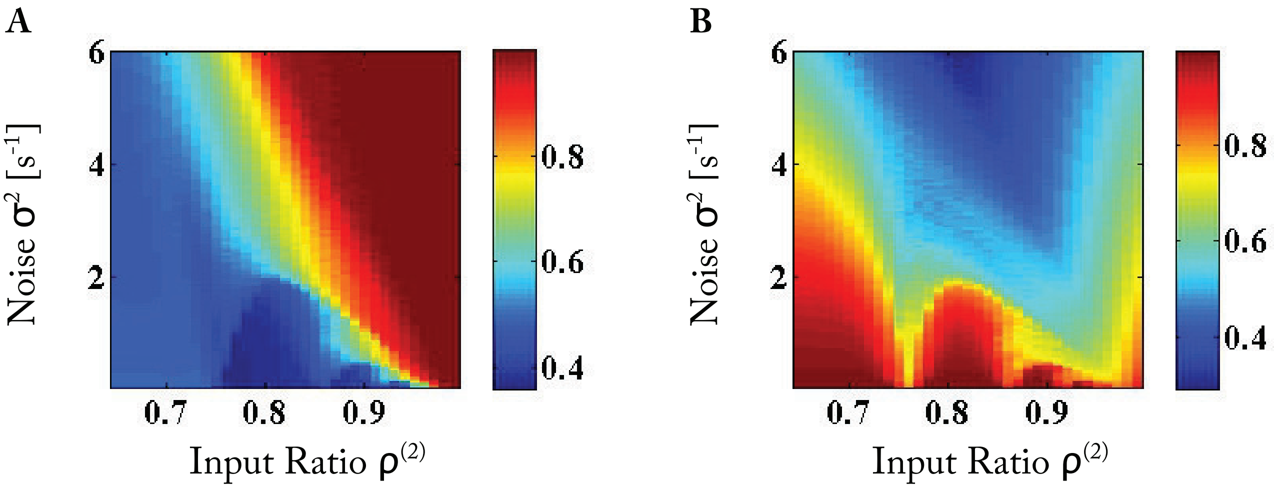

FIG. S6. Correlations between rhythms and between individual oscillators respond oppositely to noise. A) The correlation $\langle \bar{V}^{(1)}, \bar{V}^{(2)} \rangle$ between the LFP of network 2 and the periodic inhibition increases with increasing noise (cf. Fig.4). B) The average of the equal-time correlations $\langle V_j^{(2)}, V_k^{(2)} \rangle$ between individual neurons j and k in network 2 decreases monotonically with increasing noise strength. Parameters as in Fig.4.
